# Fungal contamination of medical masks among forensic healthcare workers in the COVID19 era

**DOI:** 10.1016/j.nmni.2023.101134

**Published:** 2023-04-26

**Authors:** Yassine Merad, Zoubir Belmokhtar, Omar Hadjazi, Malika Belkacemi, Derouicha Matmour, Zakaria Merad, Adila Bassaid, Ouziane Megherbi

**Affiliations:** a Central Laboratory, Parasitology-Mycology, Hassani Abdelkader Hospital, Diilali Liabes University of Medicine, Sidi Bel Abbes, Algeria; b Diilali Liabes University of Environmental Sciences, Sidi Bel Abbes, Algeria; c Forensic Department, Hassani Abdelkader Hospital, Diilali Liabes University of Medicine, Sidi Bel Abbes, Algeria; d Hemobiology Department, Hassani Abdelkader Hospital, Diilali Liabes University of Medicine, Sidi Bel Abbes, Algeria; e Therapeutic Chemistry Department, Abdelkader Hospital, Diilali Liabes University of Medicine, Sidi Bel Abbes, Algeria; f Pathology Department, Hassani Abdelkader Hospital, Diilali Liabes University of Medicine, Sidi Bel Abbes, Algeria; g Parasitology-Mycology Department, Mustapha Bacha Hospital, University of Algiers, Algeria; h ENT Department, Hospital, Diilali Liabes University of Medicine, Sidi Bel Abbes, Algeria

**Keywords:** Mask contamination, Facemasks, Covid-19 protection, Fungal contamination, Forensic workers

## Abstract

**Background:**

Medical masks are widely used in health care settings to protect healthcare workers from respiratory infections, particularly in the context of the recent Covid-19 disease.

**Methods:**

A cross-sectional study of 52 used masks collected from 52 forensic healthcare practitioners was conducted to culture for fungal isolation and identification. A study of fungal contamination was conducted by making an impression of the mouth mask cut piece on Sabouraud agar for selective isolation; each health worker completed a questionnaire, which included age, sex, type of mask, and duration of mask use.

**Results:**

Twenty five of the 52 used masks tested positive for fungal contamination (48,08%). A total of 44% of the contaminated masks belong to health workers between the ages of 21–30 years. Surgical masks (80%), KN95 (8%), and N95 (4%) were the most contaminated protective devices. Usage duration of 1–2 h was associated with 4% of fungal contamination, while a usage duration of 5–6 h was associated with 36% of fungal contamination. *Alternaria* sp (32%), *Penicillium* sp (20%), *Aspergillus* sp (16%) were the most predominant isolated fungi discovered on the inside areas of the masks.

**Conclusion:**

Because fungi are known to cause allergies and serious adverse health effects following recommendations to properly wear a medical mask is critical to preventing fungal contamination, especially among health care workers who are wearing the same mask for a long period during the pandemic.

## 1 Introduction

Because healthcare workers (HCws) are particularly vulnerable to Sars-Cov-2 exposure, wearing a facemask has become essential in the health care setting. According to the CDC guidelines, a surgical mouth mask is a personal protective barrier [1], and HCWs must wear them while performing their duties. Fungi are ubiquitous microorganisms that can be found in all parts of the world and can cause a wide range of diseases. Fungi that cause disease can enter the body through wounds, cuts, and burns, as well as the respiratory route. In addition to rhinitis, allergic bronchopulmonary mycosis, allergic fungal sinusitis, and asthma, fungus exposure has been linked to a number of other illnesses, the majority of which affect the nails or skin, causing rashes of skin conditions [2]. Manuscript (without author details) Click here to view linked References Various fungi including *Alternaria* sp*, Cladosporium* sp*,* and *Penicillium* sp [2], are traditionally associated with decay in an outdoor environment. Morbidity rates associated with fungal infections are a major public health concern. Medical masks were originally developed to filter droplets containing microorganisms expelled from the mouth [3]. In the health care setting, the most commonly worn are either the N95 mask or the surgical mask. This preliminary study aims to assess the fungal contamination of the internal part of facemasks used by forensic HCWs. Materiel and methods This cross-sectional study was carried out for one month (April 2020) in the forensic department of the university hospital of Sidi-Bel-Abbès, Algeria, to estimate the fungal contamination on used masks worn by the forensic HCWs. The subjects were recruited voluntarily for the study. An epidemiological survey was conducted during contact screening to collect information on all HCWs participants wearing masks, including gender, age, comorbidities, type of mask used, and duration of wearing. The subjects were permitted to carry out their normal work activities, but eating and physical exertion were not permitted. The study of fungal contamination was carried out by making an impression of the mouth mask cut piece on Sabouraud agar for selective isolation, blank medium were used to eliminate an environmental fungal contamination All samples were inoculated on SDA medium and incubated at 30°C for 7 to 14 days, to allow filamentous fungi and yeasts to grow. Identification of fungal contaminant species was based on macroscopic and microscopic features of growing colonies. Data were managed and analyzed using statistical software 17.0 (SPSS, Inc., Chicago, IL) Results A total of 52 participants were recruited, with 10 males and 42 females (Table 1). Out of the 52 used masks collected, 25 were positive for fungal presence, the overall fungal contamination of used masks is 48,08%, and the majority of contaminated masks belong to males (80%). The age distribution is shown in Table 1.

**Table 1:** Prevalence and factors associated with fungal contamination of facemasks.

	Variable	fungal-	fungal +	%
Gender	Female	5	5	20,0%
	Male	22	20	80,0%
Age	21–30	12	11	44,0%
	31–40	11	9	36,0%
	41–50	3	3	12,0%
	51et +	1	2	8,0%
Type of mask	Surgical mask	27	22	88,0%
	KN95	0	1	4,0%
	N95	0	2	8,0%
Mask duration usage	1–2	4	1	4,0%
	3–4	9	8	32,0%
	5–6	3	9	36,0%
	7 et +	11	7	28,0%

Fungal contamination of used masks was demonstrated on the inside area of the used mask. The prevalence of fungal masks carriage was determined between the ages of 21 and 30 years old, as summarized in Table 1, and as further displayed in Table 1, the prevalence of masks fungal carriage is significant among young health workers. Table 2 shows the different fungi isolated from the medical mask, with *Alternaria* sp (32%), *Penicillium* sp (20%), *Aspergillus* sp (16%) being the most common.

**Table 2:** Fungal species isolated from used masks Identification.

Identification	N	%
*Alternaria* sp	8	32,0%
*Penicillium* sp	5	20,0%
*Aspergillus flavus*	2	8,0%
*Aspergillus niger*	2	8,0%
*Trichophyton mentagrophytes*	2	8,0%
*Lichtheimia* sp	1	4,0%
*Candida non albicans*	1	4,0%
*Rhodotorula* sp	1	4,0%
*Fusarium* sp	1	4,0%
*Cladosporium* sp	1	4,0%
*non identified*	1	4,0%
Total	25	100,0%

## 2 Discussion

This study was done during the pandemic of Covid-19 (Sars-Cov-2). Sidi Bel Abbes HCWs were advised to wear facemasks at work. Because mask protection is the most effective device against the Sars-CoV-2 pandemic, mask reuse and extended use are common in many parts of the world, particularly during outbreaks and pandemics [4,5]. Furthermore, due to mask shortage, patients were compelled to wear the same mask for several days. According to studies, proper use of medical masks and adherence to hygiene measures are associated with lower microorganism contamination. Fungal infections can occur anywhere there is warm, moist, and creased skin. The acceptance of a mask is influenced by the air, temperature, and humidity inside the mask [6]. The majority of infections are caused by inhaling droplet nuclei ranging from 1 to 5 μm [7,8]. The current study found that the high fungal contamination on the inside area of the used surgical masks was most likely related to mask-wearing duration, environmental contamination, or HCWs habits (handling masks in white coat pockets or their instrument trays). Fungi diseases are a persistent threat, with serious diseases caused by filamentous fungi constantly emerging or re-emerging. Numerous fungi were isolated from the used masks, implying that airborne fungi accumulated on the used masks [3]. The high prevalence of masks fungal carriage among young health workers can be attributed to the occlusive effect of the mask, to the physical activity, and high breathing rate which, creates a humid environment. The stress of working with dead bodies from Covid-19 is an additional source of high breathing rate in the forensic department, additionally, the resulting sweat can make the mask wet more quickly, making breathing difficult and promoting the growth of microorganisms. It is important to remember that in such situations, HCWs are more likely to wear masks for an extended period of time and tend to re-use them, particularly in developing countries. This suggests a higher risk of masks contamination. Nonetheless, the difference in protective efficacy between facemasks and N95 respiratory is undeniable. These differences can be attributed to different material properties of the masks, such as lower air permeability, and water vapor permeability in the N95 respirator [9], moreover, N95 masks are more uncomfortable to wear compared to surgical masks and this may affect the contamination levels noted.

After 3 h of wearing, we noticed an increase in fungal contamination, which we believe was caused by temperature and humidity fluctuations. Wearing a facemask for a certain period may raise the temperature inside the oral cavity by reducing heat convection and evaporation from the mouth to the surrounding environment [10]. A fungal infection can thrive in the moist environment provided by the mask. During an outbreak of infectious diseases, like SARS and Sars-CoV-2 HCWs may be required to wear a mask for several hours, if not a day [10]. Furthermore, HCWs are wearing masks during their activities and even at home to avoid familial contamination, such reactions are reminiscent of behavior during the 2003 SARS outbreak, when not only the general public, but even close family members were afraid of being infected by HCWs exposed to the disease [11]. In addition, unsanitary re-use of face masks, particularly during the prevailing hot and humid weather was responsible for increasing the risk of Mucormycosis in India [12], and the genera Lichtheimia recovered in this study is considered as a proven Mucormycosis pathogen among immunosuppressed individuals. Additionally, an invasive mucormycosis due to *Lichtheimia* sp following tooth implants has already been reported in our medical center [13].

In all epidemiologic studies, mold exposure and dampness are considered together, with dampness is defined as sufficient moisture on or a substrate to support fungal growth [2]. The humidity content of the mask air was only significant when the mask air was warm [6]. Moreover, microorganisms can spread easily in spaces such as hospital wards. Furthermore, keeping the mouth mask in white coat pockets or instrument trays, as well as regular handling of the masks for adjustments and during a conversation, can be considered risk factors for masks contamination [14]. According to previous studies, the majority of the organisms isolated from the mouth-masks in this study were potentially pathogenic [12,[15], [16], [17]], 32% of all the colonies were identified as Alternaria, which are also considered to be outdoor airborne fungi. Along with the four most abundant isolated species, *Penicillium* sp*, Aspergillus flavus, Aspergillus niger*, and *Trichophyton mentagrophytes*. Likewise, Alternaria, and Cladosporium are known to cause allergies [2]. A link between fungi exposure and the risk of respiratory symptoms has already been established, regardless of sensitization [2]. Furthermore, in a study of adults with asthma, 76% of patients with multiple hospital admissions had at least one positive fungal skin test to Aspergillus, Alternaria, Cladosporium, and Penicillium genera [2], which were recovered in our study, and are considered as common outdoor fungi.

Recent studies in our hospital are suggesting a possible infectious role of these species among HIV, psychiatric and oncology inpatient [[17], [18], [19], [20]]. Moreover, most of these fungi were also isolated from patients wearing earmolds, and cockroaches trapped in several medical departments [15,21].

In several studies, the use of face masks has been linked to acne caused by an accumulation of *S. aureus*, moreover, acne, rosacea, and seborrheic dermatitis conditions can either be more prevalent or worsened [22,23]. *Trichophyton mentagrophytes* is linked to tinea and sycosis, and this finding may indicate a potential skin disorder in HCW mask wearers, which is supported by previous research [23]. It is also possible that face masks induce abnormalities of both local microbiota and skin barrier permeability [23]. Most common fungi, however, are frequently found indoors because they enter through open doors and windows and can be carried inside. While wearing a medical mask may have little or no clinical impact, however, it can be a source of fungal contamination in health care settings, particularly among immunosuppressed patients. What is still unknown is how much exposure is required (amount and duration), whether there is a dose-response, whether specific fungi genera are responsible for the effect, and whether intervention to reduce exposure would prevent health-related adverse effects. The microbial counts in indoor air are affected by a variety of factors, including velocity, humidity, temperature, ventilation, the number of occupants, people’s activities, particulate matter or dust concentration, and the outdoor air quality [[24], [25], [26]]. To reduce the fungal contamination on the used masks, hospital environments should be improved, particularly microbial air quality in working wards. Wards in the unit should be cleaned regularly to prevent the growth of moisture-borne pathogens. To prevent microbial growth, avoid wet surfaces, keep relative humidity levels below 70%, use effective particulate filtration, good housekeeping, and operate and maintain HVAC system properly [27,28].

There are some limitations in this study that must be addressed, particularly the small sample size. Another issue is the source of fungal contamination, mycological results were based solely on the mask contamination not on the environment contamination, which may have probably influenced the findings. This would be more accurately estimated if PCR technique were applied on both masks and environment. In addition, in addition, we didn’t investigate mask-wearing behavior at an individual level. More research is needed to evaluate the efficacy of mask-wearing in various settings. We are unable to confirm the source of contamination because other factors associated with fungal exposure may also influence any observed health effects. The Ethics Committee of the institution approved this study, and all participants provided informed consent. In this paper, we present an observational cross-sectional analysis of data collected from HCWs masks in April 2020.

## 3 Conclusion

In this study on fungal masks contamination, a wide variety of fungi were recovered. Wearing a mask for extended periods, especially on hot, humid summer days, can thus promote fungal contamination. The interesting findings in our study probably could be explained by the fact that wearing the same facemask for an extended period may promote fungal overgrowth. The establishment and implementation of health and safety standards, as well as monitoring of the work environment, should be considered.

## 4 Learning points

What is already known about this subject?

• Healthcare workers are on the front lines of the fight against covid19 pandemic. They are frequently at high risk of infection in the workplace and must wear mask protection for a long time.
• When assessing the risk of microbial mass contamination in healthcare workers, it is critical to understand when and how masks become infected in the hospital, whether by the environment or health workers.
What this study adds:
• To the best of our knowledge, this is the first study on fungal contamination of face masks among HCWs so far.
• Healthcare workers may underestimate the risk of dermatophytes and airborne fungi and disregard protective measures during mask usage.
• Mask fungal carriage is significant and may have a substantial impact on fungal transmission in hospital settings. Although it was not a large-scale study, our findings may provide some hints on this issue.

What impact this may have on practice or policy:

• Our findings may raise worker awareness on the importance of using additional protective measures when wearing masks.
• Strengthening administrative control measures, improving mask quality, working environment, and limiting the duration of mask‘s wearing could have a significant impact on reducing its fungal load and some fungal adverse effects on health such as rhinitis.

## CRediT authorship contribution statement

**Yassine Merad:** contributed in collecting, Formal analysis, the results, Writing – review & editing. **Zoubir Belmokhtar:** contributed in collecting, Formal analysis, the results, Writing – review & editing. **Malika Belkacemi:** contributed in collecting, Formal analysis, the results, Writing – review & editing. **Derouicha Matmour:** contributed in collecting, Formal analysis, the results, Writing – review & editing. **Zakaria Merad:** contributed to writing, Writing – review & editing. **Adila Bassaid:** contributed to writing, Writing – review & editing. **Ouziane Megherbi:** contributed to writing, Writing – review & editing.

## Declaration of competing interest

We have no conflict of interest to declare.
